# Are All Writers Men of Genius?

**Published:** 1892-01-23

**Authors:** 


### The Doctor's Armchair.

### ARE ALL WRITERS MEN OF GENIUS?

Professor Cesare Lombroso insists that we shall accept his dictum that genius and insanity are twin brothers. He is particularly anxious to convince the dwellers in the British Isles, and other speakers of the English tongue, of the scientific soundness of this conclusion. So far, however, as appearances go at present the result of the Professor's labours seems to be that the more he persuades the less persuasive he becomes. His book, just published in London by Walter Scott, reiterates what he has several times previously said, and brings the reader no nearer to the point of conviction than he was before he read it.

It is necessary to join issue with the learned Professor at the very outset of his argument. He takes it for granted that great writers are men of the greatest genius; and that if a man have genius he will show it by writing books, and practically in hardly any other way. To that view of the subject we take the most decided objection. There have been thousands of men of genius in the world who have never either written books or thought of writing them; and there have been thousands of men and women in the world who have written books, but who have not had one single spark of genius in their composition.

Let any intelligent man take a glance round among his own contemporaries, public and private. If he does this with candour he will be struck by the fact that the men and women of undoubted genius among those contemporaries are exceedingly few in number. But he will see, on the other hand, that the writers of books and newspapers may be counted by tens of thousands. What is it that makes a man or a woman wish to write a book or a newspaper article? Is it genius, always genius, and nothing but genius? Let us appeal to the editors of newspapers and to hard-driven publishers' readers. To that jury we would address this simple question: "Is it possible to find in all the world a more absolutely inconsequent set of people than many of those who send articles to newspaper editors or books to publishers?" The gift of writing in itself is nothing more than a mere power of loquacity. When a person, especially of the female sex, finds herself endowed with an unusual amount of courage—to call it by its most respectable name—together with a gift of speech, she forthwith thinks herself a born genius, and scribbles off an article at a galloping pace, and sends it, in the breathless excitement of anticipated triumph, to the nearest newspaper office.

This is genius, or, at any rate, it is unabashed precocity, which Professor Lombroso seems to consider identical with genius. Men of genius, affirms the Professor, are mostly small in stature, and are often rickety, lame, hunch-backed, or club-footed. They are pale, bloodless, and fleshless; they suffer from diseases of the brain and head; they stutter, they are left-handed, they are somnambulists, they are vagabonds, they marry, but do not have children; in a word, from the point of view of plain sense and practicality, they are generally contemptible.

If one had the wand of a magician, one would call up the re-embodied ghosts of thousands of the men of practical, as distinguished from mere quill-driving genius, who have played their part in the world's story —the soldiers, the sailors, the travellers, the builders, the rulers of states, the architects, the priests, the tillers of the soil, the Nimrods, the Alexanders, the Columbuses, the Antonies, the Wellingtons, the Washingtons, together with a few sane writers such as Homer, Cæsar, Shakespeare, Burke, Tennyson—these, and ten thousand like them, would constitute a vast army of well-built, firmly knit, solid, sober, and sane men. Place such as these over against Professor Lombroso's rickety, skinny, small-headed, and neurotic shriekers and chatterers, and the latter will appear absolutely ludicrous and contemptible in comparison.

A good many men of genius have given themselves to the writing of books during the world's history. But where one man of genius has given himself up to writing, it is probable that ten thousand of as great or greater genius have devoted themselves to what they considered the more immediately useful and important duty of doing the world's practical work. One may admire Heine and Marie Bashkirtseff for a kind of firework brilliance which they have possessed, but there are thousands of solid farm labourers who are to-day ploughing and tilling the soil of our native land, anyone of whom, for practical sense—true genius—is worth a whole regiment of Heines, Schopenhauers, or Bashkirtseffs. One ought to remember that the reason why the world is filled with the fame of writers rather than with that of practical men is that other writers are in possession of all the world's trumpets, and that naturally they blow loudest when they are praising their own kind.

Let anyone but give himself the trouble to read through the reviewer's columns of our daily and weekly newspapers for a few weeks. He will there find the novels and other published works of all kinds of inconsequent and silly persons seriously reviewed, although it is quite evident that the reviewer himself is absolutely nauseated with the stupidity and worthlessness of the things he is obliged to speak of seriously and with a certain kind of polite respect. But if those absurd writers had devoted their industry to some other work than that of writing books—say, for example, to the work of making dumplings, or of darning stockings, or of cutting out coats, or of dibbling broad beans for the next summer's crop—does anyone suppose for a moment that the newspapers or any other part of the great world would have taken the least notice of them and their doings? But because, like the London sparrow, they can chatter, and because they can write sentences which read more or less like English, and because they have the assurance that there are people in the world silly enough to read what they may choose to write, therefore they incontinently rush into print; therefore also they are seriously reviewed; and what is to prevent them from concluding that they are persons of genius if they so please?

Whatever else be true of genius, this, at any rate, is true, that the most distinguishing of all its marks is sanity. The insane genius is like the flawed Sheffield blade—"a waster." He is a second-rate man: twin brother to the fool; and bears about him a thousand marks of his kinship. Men whose genius and sanity are of the second or third order may do considerable things in the world if they work industriously and persistently for many years; but when our Father Time has really great words and deeds to show to his children he always speaks by the mouth and works by the hand of men who are sober and sane.

